# Minimal important difference and patient acceptable symptom state for the Numerical Rating Scale (NRS) for pain and the Patient-Rated Wrist/Hand Evaluation (PRWHE) for patients with osteoarthritis at the base of thumb

**DOI:** 10.1186/s12874-022-01600-1

**Published:** 2022-04-29

**Authors:** Susanna Stjernberg-Salmela, Teemu Karjalainen, Joona Juurakko, Pirjo Toivonen, Eero Waris, Simo Taimela, Clare L. Ardern, Teppo L. N. Järvinen, Jarkko Jokihaara

**Affiliations:** 1 Department of Hand Surgery, University of Helsinki and Helsinki University Hospital, Helsinki, Finland; 2 Finnish Centre of Evidence-Based Orthopedics (FICEBO), University of Helsinki and Helsinki University Hospital, Helsinki, Finland; 3 Central Finland Central Hospital, Jyväskylä, Finland; 4 Central Finland Health Care District, Jyväskylä, Finland; 5 Department of Orthopaedics and Traumatology, University of Helsinki and Helsinki University Hospital, Helsinki, Finland; 6 Department of Family Practice, University of British Columbia, Vancouver, Canada; 7 Department of Hand and Microsurgery, Tampere University Hospital, Kuntokatu 2, 33520 Tampere, Finland; 8 Faculty of Medicine and Health Technology, Tampere University, Arvo Ylpon katu 6, 33520 Tampere, Finland

**Keywords:** Minimal important difference, Patient acceptable symptom state, Patient reported outcome measures, Treatment outcome, Osteoarthritis, Wrist, Hand

## Abstract

**Background:**

The Numerical Rating Scale (NRS) and Patient-rated wrist/hand evaluation (PRWHE) are patient-reported outcomes frequently used for evaluating pain and function of the wrist and hand. The aim of this study was to determine thresholds for minimal important difference (MID) and patient acceptable symptom state (PASS) for NRS pain and PRWHE instruments in patients with base of thumb osteoarthritis.

**Methods:**

Fifty-two patients with symptomatic base of thumb osteoarthritis wore a splint for six weeks before undergoing trapeziectomy. NRS pain (0 to 10) and PRWHE (0 to 100) were collected at the time of recruitment (baseline), after splint immobilization prior to surgery, and at 3, 6, 9 and 12 months after surgery. Four anchor-based methods were used to determine MID for NRS pain and PRWHE: the receiver operating characteristics (ROC) curve, the mean difference of change (MDC), the mean change (MC) and the predictive modelling methods. Two approaches were used to determine PASS for NRS pain and PRWHE: the 75^th^ percentile and the ROC curve methods. The anchor question for MID was the change perceived by the patient compared with baseline; the anchor question for PASS was whether the patient would be satisfied if the condition were to stay similar. The correlation between the transition anchor at baseline and the outcome at all time points combined was calculated using the Spearman’s rho analysis.

**Results:**

The MID for NRS pain was 2.5 using the ROC curve method, 2.0 using the MDC method, 2.8 using the MC method, and 2.5 using the predictive modelling method. The corresponding MIDs for PRWHE were 22, 24, 10, and 20. The PASS values for NRS pain and PRWHE were 2.5 and 30 using the ROC curve method, and 2.0 and 22 using the 75th percentile method, respectively. The area under curve (AUC) analyses showed excellent discrimination for all measures.

**Conclusion:**

We found credible MID estimates for NRS and PRWHE (including its subscales), although the MID estimates varied depending on the method used. The estimates were 20-30% of the range of scores of the instruments. The cut-offs for MID and PASS showed good or excellent discrimination, lending support for their use in future studies.

**Trial registration:**

This clinimetrics study was approved by the Helsinki University ethical review board (HUS1525/2017).

**Supplementary Information:**

The online version contains supplementary material available at 10.1186/s12874-022-01600-1.

## Background

The thumb accounts for about half of the function of the hand and plays a key role in manipulating objects [1]. Functionally, the most important joint of the thumb is the trapeziometacarpal (TMC) joint with a wide range of motion [1]. The TMC joint is susceptible to osteoarthritis leading to pain and disability in activities of daily living [2]. Osteoarthritis of the TMC joint affects 6% of men and 7% of women over the age of 50, and up to 33% of men and 39% of women over the age of 80 [3, 4].

The Visual Analogue Scale (VAS) and the Numeric Rating Scale (NRS) are the most frequently used unidimensional measures for hand pain. Other outcome measures, such as the Patient-Rated Wrist/Hand Evaluation (PRWHE) and the Australian Canadian Osteoarthritis Hand Index (AUSCAN), are typically used to assess hand function and disability [5–7]. Validated patient-reported outcome measures (PROM) are essential for assessing the signs and symptoms of a hand condition and the effects of different interventions. However, the scores of these instruments may be difficult to interpret. To facilitate the interpretation of an outcome instrument, two established concepts, the minimal important difference (MID) (sometimes called the minimal clinically important difference) and the patient acceptable symptom state (PASS) were developed [8, 9].

The MID concept was introduced over 30 years ago and became a gold standard in evaluating the clinical relevance of trial results [8]. It represents the smallest difference in a score that a patient perceives as a change (either beneficial or harmful) regarding the concern that is under investigation [8], or the smallest difference in outcome that patients or informed proxies consider important, likely leading the patient or the treating clinician to consider a change in the strategy of treatment [10]. MID can be used to compare the outcomes in two groups of patients at a certain point in time (e.g., in a trial or a meta-analysis) or to compare the change in outcome in one group (or patient) as a function of time [11].

Patient acceptable symptom state (PASS) was introduced more recently, and thus, its use is less common in musculoskeletal research [12]. PASS represents the cut off value for symptoms beyond which patients consider themselves well [13]. It is a value based on patients’ opinion of the overall state of the symptoms. Achieving PASS can be considered the goal of treatment: it describes the state at which patients feel their condition has improved to a level that they are comfortable with [9]. PASS can also be used to calculate the proportion of patients achieving a satisfactory clinical state, and it may be less dependent than the MID on the clinical situation at baseline**.** Predefined PASS values may prevent manipulation of the cut off in responder analyses and improve the interpretability of clinical trial findings [14, 15].

The aim of this study was to define the MID and PASS thresholds for NRS pain and PRWHE in patients undergoing surgery for base of thumb osteoarthritis.

## Methods

Participants were recruited at the Central Finland Central Hospital, the Tampere University Hospital and the Helsinki University Hospital between August 2017 and November 2018. The study protocol was approved by the Ethical Review Board of Helsinki University Hospital (HUS 1525/2017) and local Institutional Review Boards.

### Design and setting

**Fig. 1:**
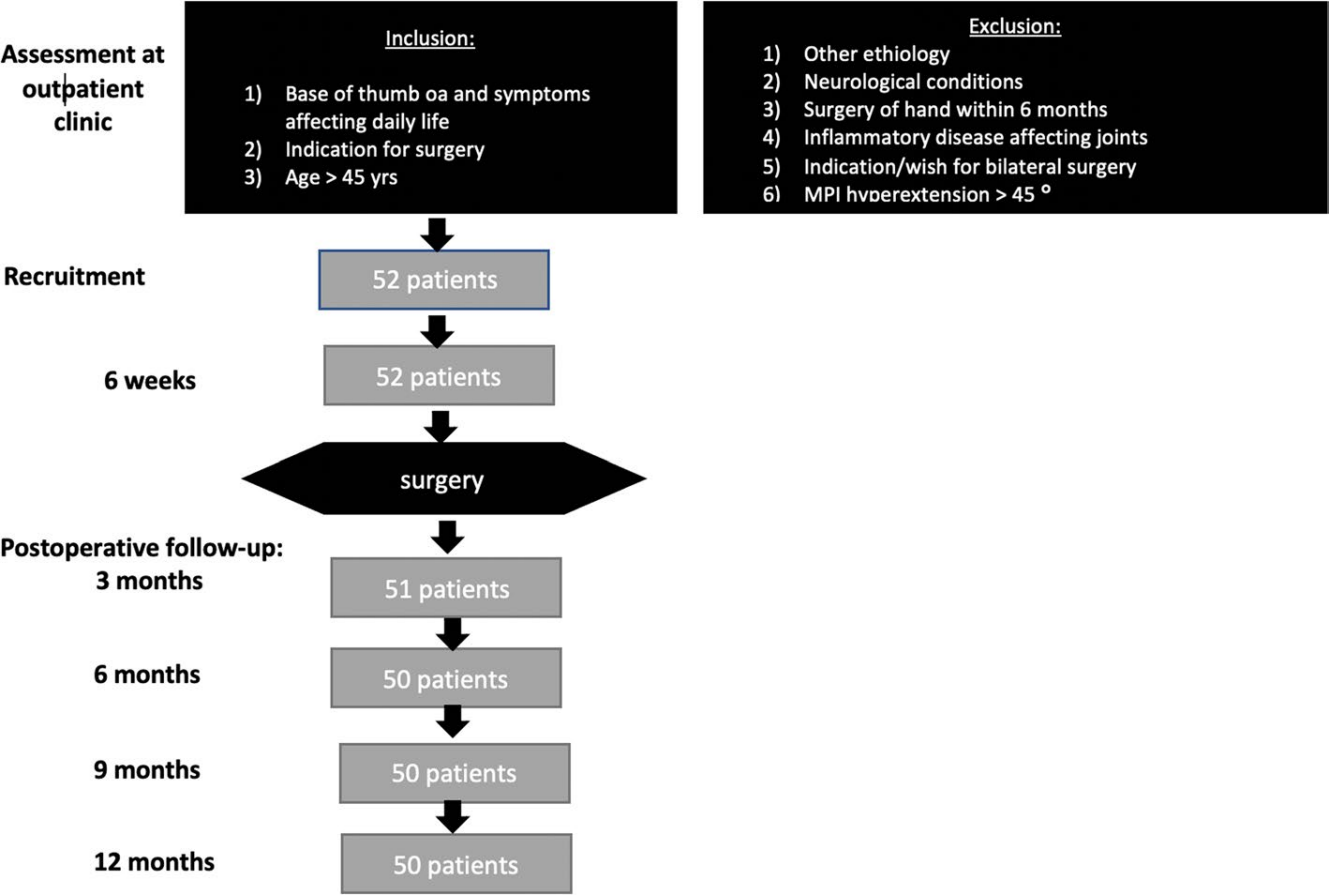
Overview of the recruitment process

This is a prospective cohort study. Three experienced hand surgeons screened all patients who were referred for surgical consultation for radiologically verified base of thumb osteoarthritis at the participating secondary or tertiary referral hospitals. Prior to recruitment, all participants were scheduled for surgery because non-operative treatment had not relieved their symptoms. The specific inclusion criteria were: 1) thumb osteoarthritis with symptoms affecting activities of daily living, 2) a present indication for surgical treatment of base of thumb osteoarthritis based on radiographic and clinical findings, and 3) age >45 years. The exclusion criteria were: 1) symptoms likely due to something other than base of thumb osteoarthritis, 2) the presence of a neurological condition that may affect hand function, 3) surgical treatment of the hand within the previous 6 months, 4) an inflammatory condition affecting joints, 5) the presence of an indication for bilateral surgery for base of thumb osteoarthritis, or 6) >45º hyperextension in the thumb metacarpophalangeal joint (zig zag -deformity). The recruitment procedure is presented in Fig. 1.

Participants were informed about the study and gave informed consent. Thereafter, all participants received a removable splint (Actimove Rhizo Forte splint, Essity AB, Stockholm, Sweden) to wear full time for 6 weeks before surgery. The intent of preoperative splinting was to provide a uniform and systematic approach to non-operative treatment, in addition to the different forms of non-operative treatment issued before referral to surgical consultation. Our approach allowed for a time point of clinical evaluation and collection of data following this 6-week period of non-operative treatment. In the surgical intervention, a simple trapeziectomy, in which the trapezium was removed completely, was performed. After the operation, the wrist and the thumb were immobilized in a thumb spica cast for 3 weeks, followed by 3-week immobilization with a removable splint.

### Baseline data and outcome measures

At baseline, we collected the following data from participants: age, sex, handedness, affected side, use of tobacco products, radiological staging of the thumb basal joint osteoarthritis (Eaton-Glickel) [16], and height and weight for calculation of the body mass index (BMI).

We collected NRS pain and PRWHE at the baseline, after 6 weeks of splinting prior to surgery, and at 3, 6, 9, and 12 months after surgery. For NRS pain, the participants were asked to rate average pain intensity in activities of daily living during the previous day by selecting a value from 0 to 10 (0 indicating no pain and 10 indicating the worst pain imaginable). PRWHE is a 15-item questionnaire, in which patients rate their level of wrist/hand pain and disability on a scale from 0 to 10 for each item. PRWHE consists of independent subscales for pain and for hand/wrist function. Both subscales are scored from 0 to 50, and thus the total score is from 0 to 100 (0 is the best and 100 is the worst possible score) [17, 18]. A higher PRWHE pain subscale score represents worse pain, and a higher PRWHE function subscale represents worse function.

As an anchor question for determining the MID, [19] participants were asked to rate the change in their condition compared to baseline on a five-step rating scale, which provided the global rating of change (GRC) [20]. The response options were: 1) much better, 2) somewhat better, 3) unchanged, 4) somewhat worse, and 5) much worse. As an anchor question for PASS, the participants were queried whether they would be satisfied with the present state of the thumb if it were to stay similar for the rest of their lives. The response options were yes or no.

### Analyses

Change in NRS pain and PRWHE scores were calculated for each patient by subtracting the score at the time of follow-up from the baseline score. We used data from all time points in the analyses, i.e., each participant provided an anchor question–outcome measure data pair at each of the five follow-up timepoints. To assess the adequacy of the anchor question, we calculated Spearman's correlation between the anchor question and 1) baseline score; 2) follow-up score; and 3) change score, and values >0.5 were considered sufficient as suggested by Devji et al. [21]. 95% confidence intervals for Spearman’s rho were derived by bootstrapping 1000 samples.

Four different approaches were used to determine the MID: 1) For the mean change method (MC) we calculated the mean value for participants who reported “somewhat better” on the GRC; 2) for the mean difference of change method (MDC) we calculated the mean difference between those who had reported “somewhat better” and those who reported “no change” on the GRC; 3) for the Receiver Operating Characteristics (ROC) curve method we dichotomized the GRC between “no change” and “somewhat better”, excluding participants who reported “somewhat worse” or “much worse” [22], and for the predictive modelling method we used logistic regression analysis as described by Terwee et al. 2021 [23]. The optimal cut point on the ROC curve was determined using the closest point to the top left corner [24]. The area under the ROC curve (AUC) and the 95% confidence interval for ROC MID values were calculated using non-parametric bootstrapping with 1000 replications [23]. AUC values between 0.7 and 0.8 indicate acceptable discrimination and values >0.8 indicate excellent discrimination [25]. 95% confidence intervals for AUC were calculated using DeLong’s method [26].

To determine the PASS, we used two methods: 1) the ROC curve, similar to the method used for the MID analysis (cut off-point discriminating between an acceptable versus a non-acceptable symptom state); and 2) the 75^th^ percentile method [27, 28]. The PASS was defined as the 75^th^ percentile score for both NRS pain and PRWHE in the distribution of the patients who considered themselves to be in an acceptable symptom state.

## Results

Fifty-two participants were recruited into the study; two did not attend the follow-up visits. Thus, we had data from 50 participants at five follow-up time points (250 outcome–anchor question pairs). We did not define MID values for all separate time points due to the low number of participants reporting no change in global rating of change at several time points. Also, as correlations between GRC and the change of the target instrument were low at some time points (supplementary appendices S1, S2 and S3), it was not reasonable to analyze MID estimates for every follow-up. Postoperative recovery in patients with osteoarthritis at the base of the thumb is a process that evolves over a long period of time, and the answer to the anchor question is likely to vary at different time points. As MID is determined using anchor-outcome score pairs, without time as a factor or covariate in the analysis, including all values at the different time points is expected to yield a more accurate MID estimate. The participant characteristics are described in Table 1.

**Table 1:** Baseline characteristics of the participants in the study

	N (%)
**Sex**	
Female	39 (75)
Male	13 (25)
**Handedness**	
Right	43 (83)
Left	6 (12)
Ambidextrous	3 (5.8)
**Affected side**	
Dominant	19 (37)
Non-dominant	27 (52)
Bilateral	6 (12)
**Smoker**	
Yes	11 (21)
No	41 (79)
**Eaton-Glickel classification**	
Stage 2	9 (17)
Stage 3	20 (39)
Stage 4	22 (42)
	Mean (Std. Deviation)
**Age, years**	62 (7.6)
**BMI, kg/m**^**2**^	28 (4.4)
**NRS pain, past day (0 to 10)**	6.5 (1.7)
**PRWHE total score (0 to 100)**	64.3 (12.6)
**PRWHE pain subscale (0 to 50)**	34.2 (6.8)
**PRWHE function subscale (0 to 50)**	30 (7.9)

The MID estimates from the ROC analysis are presented in Table 2. In the ROC analysis (for all time points combined) MID for NRS pain showed good (AUC>0.8) discrimination and for the PRWHE pain and function subscales, as well as the PRWHE total score, showed excellent discrimination (AUC>0.9) [25].

**Table 2:** The MID estimates from the ROC analysis

Outcome measure	MID	Sensitivity	Specificity	AUC (95 % CI)
NRS pain	2.5	0.79	0.82	0.84 (0.75 to 0.93)
PRWHE total score	10	0.91	0.86	0.93 (0.89 to 0.97)
PRWHE pain subscale	7.5	0.87	0.91	0.93 (0.89 to 0.96)
PRWHE function subscale	6.0	0.86	0.82	0.90 (0.85 to 0.96)

NRS pain from 0 to 10 (0 = no pain), PRWHE total score from 0 to 100, PRWHE pain and function subscales from 0 to 50 (0 = optimal situation)

Deteriorated GRCs were excluded from the analysis, NRS pain from 0 to 10 (0 = no pain), PRWHE total score from 0 to 100, PRWHE pain and function subscales from 0 to 50 (0 = optimal situation).

MID values from the mean difference of change method, mean change method and predictive modelling method were higher than the MID values calculated using the ROC curve method for PRWHE pain and PRWHE total (Table 3). The MID values for NRS pain and the PRWHE function subscale were comparable for all four methods. The MID values ranged from 20% to 25% of the scale of NRS pain and 10% to 26% of the scale of the PRWHE total score or subscale.

**Table 3:** The MID estimates from the mean difference of change (MDC), mean change (MC), ROC and Predictive modelling method

Outcome measure	MDC	MC	ROC	Predictive
NRS pain	2.0 (0.8 to 3.2)	2.8 (2.1 to 3.5)	2.5 (2 to 2.9)	2.5 (2.0 to 2.9)
PRWHE total score	22 (16 to 28)	24 (20 to 28)	10 (8 to 23)	20 (14 to 24)
PRWHE pain subscale	12 (8 to 15)	13 (10 to 16)	7.5 (3 to 8)	10 (7 to 12)
PRWHE function subscale	10 (6 to 14)	11 (9 to 13)	6.0 (4 to 16)	10 (7 to 13)

Values are MID with 95 % CI

The correlation between the GRC and the post scores and the change of the target instrument (reflecting reliability of the MID values) were at an acceptable level (>0.5) [29] for the outcomes at all time points combined (Tables S1 and S2 in the supplementary appendix). NRS pain and PRWHE total score showed strong correlation with the GRC, both being 0.72. The correlation of the PRWHE pain and function subscales with the GRC were 0.73 and 0.69, thus indicating that the MID estimates were credible for these measures at all time points [30]. The correlation between GRC and the change of the target instruments showed strong correlation for PRWHE total score and subscales for pain and function, being -0.74, -0.71 and -0.70, respectively, and for NRS pain, being -0.55 (Table S2 in the supplementary appendix). The correlations between GRC and the target instrument baseline scores showed acceptable values for all target instruments at 3- and 6-month time points, ranging from -0.30 to -0.08 (Table S3 in the supplementary appendix), but not at later time points, implying that with passing time, the participants in the study may find it more difficult to relate their current clinical situation to the situation at baseline.

PASS values derived using the ROC curve method were slightly higher than those derived by the 75th percentile method, but both methods yielded values that were 20%-25% of the NRS pain range of scores, and 20%-34% of the range of the PRWHE total score or of its subscales. The AUCs implied excellent discrimination for all outcome measures (Table 4).

**Table 4:** PASS estimates

Outcome measure	75^**th**^ percentile method	ROC method			
	PASS	PASS	Sensitivity	Specificity	AUC (95% CI)
NRS pain	2	2.5	0.82	0.91	0.92 (0.89 to 0.95)
PRWHE total	22	30	0.88	0.90	0.95 (0.93 to 0.97)
PRWHE pain	13	17	0.87	0.93	0.96 (0.93 to 0.98)
PRWHE function	10	12	0.85	0.87	0.93 (0.90 to 0.96)

NRS pain 0 to 10 (0 = no pain), PRWHE pain and function 0 to 50, and PRWHE total 0 to 100 (0= optimal situation)

## Discussion

We used four different established methods to estimate the MID value and two different methods to estimate the PASS value for two outcome instruments commonly used when studying patients with osteoarthritis at the base of the thumb. The MID and PASS estimates for both NRS pain and PRWHE (including its subscales for pain and function) ranged from roughly 20 to 30% of the scale of the instrument depending on the method used. Most importantly, the obtained cut-offs for both instruments showed good or excellent discrimination – essentially, the MID cut-offs distinguishing “those with at least some improvement” from “those with no improvement” and the PASS cut-offs “the satisfied” from “the unsatisfied”. These findings suggest that the MID and PASS estimates for NRS pain and PRWHE found in this study may be sufficiently robust for drawing interferences on the clinical meaningfulness of different studies on patients with base of thumb osteoarthritis using these outcome instruments.

High internal validity is an obvious strength of our study: We used four different, established methods to estimate the MID value and two methods to estimate the PASS value, and the study was conducted by a staff with ample clinical research background. Experienced staff (hand surgeons and assisting personnel) ensured uniform indications for surgery, the homogeneity of the study population, and strict adherence to the pre-specified study protocol (2/52 or 4% loss to follow-up). In addition, we consider our anchor question for PASS to be sufficiently clearly defined for assessing the patients’ overall satisfaction.

Our study also had limitations. The relatively small (*n*=50) study population compelled us to combine all follow-up time points, and accordingly, we could not assess whether the MID and PASS estimates vary as a function of time.

The results of our study are comparable with the findings in two recent studies reporting estimates for the minimal important change (MIC) and PASS for NRS pain for patients undergoing proximal interphalangeal (PIP) joint arthroplasty [31], and estimates for MIC, MID and PASS in patients undergoing surgery for symptomatic base of thumb osteoarthritis [32]. In the first study, a PASS estimate of 1.5 for NRS pain at rest and 2.5 for NRS pain during activities were reported. The same study reported a MIC value of -1.2 for NRS pain at rest and -2.8 for pain during activities at one-year postoperative follow-up [31]. In the latter study, the postoperative MIC estimate for pain at rest and pain during activities and were 1.9 and 3.9, respectively. The MID estimates for NRS pain at rest and pain during activities were 1.4 and 1.0, and the PASS value were 1.5 and 2.5, respectively [32]. However, direct comparison of the NRS pain estimates in our study with the NRS pain estimates in the above-mentioned studies, is difficult, as our study evaluated overall NRS pain whereas NRS pain in the other studies was evaluated at rest and during activities.

MID and PASS for PROMs used in hand surgery have been sparsely studied, particularly for patients with osteoarthritis at the base of the thumb. MID estimates have, however, been defined for diverse populations of patients with hand surgery [33, 34]. For PRWE, MID estimates have been investigated in patients who received non-surgical treatment for hand and upper extremity problems [35, 36], for patients undergoing ulnar shortening osteotomy [37] and for patients with various hand and wrist injuries [7]. PASS estimates for AUSCAN and other general- and disease-specific PROMs have been determined for patients with hand osteoarthritis [38, 39] and for QuickDASH for patients after open carpal tunnel release [40]. Another study estimated MIC for QuickDASH in patients receiving surgical treatment for symptomatic osteoarthritis at the base of the thumb, using an anchor based-method and a distribution-based method [41]. We did not identify studies determining PASS values for PRWHE in patients with hand surgery.

Regarding the determination of MID and PASS estimates more broadly, our findings are well aligned with the existing understanding that there is a variability in the MID estimates between different methods and different outcome measures [42]. The higher MID values using the MDC method or MC method compared with the results of the ROC method demonstrate the uncertainty regarding cut off-values of a continuous variable, whereas the estimates derived by the predictive modelling method were close to results of the ROC method for NRS pain and close to the results of the MDC and the MID methods for PRWHE and its subscales. The predictive modelling method yielded similar values as ROC when the variation of the score of those who improve and those who do not is similar. The benefit of the predictive modelling method is that it yields estimates that are more precise than estimates derived by the ROC method [43]. The MC method is sensitive to random variation when the sample is small, and it misclassifies about half of the participants due to the use of the mean value. The MDC method, on the other hand, is sensitive to the mean value of the people who did not experience a change, which can be small and therefore subject to a large random variation. The benefit of the ROC method is that it identifies the optimal cut off for discrimination, i.e., it yields a value that misclassifies the least in a given sample.

When evaluating the results of operative treatment in patients with base of thumb osteoarthritis, the clinical applicability of MID is that it can be used 1) to assess the relevance of between group differences, if comparing different surgical techniques, and 2) to estimate the proportion of patients who noticed a change in their condition and were satisfied with the result (i.e. to identify the proportion of the patients for whom operative treatment could be considered worthwhile [27]). PASS can be applied only for the latter purpose, but it may better reflect the aims of surgery, i.e. not those who could perceive a small change, but those who achieved a satisfactory level of symptoms. The cut offs are not applicable on an individual level, as they vary between individuals. Instead, direct questions can be used when assessing individual patients in clinical practice.

Future research could investigate the effect of time on MID and PASS estimates and examine the underlying causes for the variation in the MID and the PASS estimates with regard to the method of analysis used.

## Conclusion

This study defined the MID and the PASS estimates for NRS pain, PRWHE total, and the PRWHE pain and function subscales, in patients who received surgery for symptomatic osteoarthritis at the base of the thumb. The MID estimates varied depending on the method used. The MID values determined in this study for all these outcome measures appear credible, as the GRC correlates well with the post score and the change on the target instrument. The cut-offs for both MID and PASS showed good or excellent discrimination, suggesting that they could be meaningful in interpreting NRS pain and PRWHE scores in studies evaluating the effect of surgery for patients with osteoarthritis at the base of the thumb.

## Supplementary Information

**Additional file 1: Table S1.** Correlations between post scores and GRC. **Table S2.** Correlations between GRC and the change of the target instrument. **Table S3.** Correlations between GRC and target instrument baseline scores.

## Data Availability

The datasets supporting the conclusions of this article are available upon request from the corresponding author Jarkko Jokihaara, MD, PhD, Department of Hand and Microsurgery, Tampere University Hospital, Kuntokatu 2, 33520 Tampere, Finland (email: Jarkko.Jokihaara@pshp.fi).

## Abbreviations

VAS: Visual analog scale
NRS: Numeric rating scale
PRWHE: Patient-rated wrist/hand evaluation
AUSCAN: Australian Canadian Osteoarthritis Hand Index
PROM: Patient reported outcome measure
MID: Minimal important difference
PASS: Patient acceptable symptom state
ROC: Receiver operating characteristic
MDC: Mean difference of change
MC: Mean change
AUC: Area under curve
GRC: Global rating of change
