# Supplementary material for: Minimal important difference and patient acceptable symptom state for the Numerical Rating Scale (NRS) for pain and the Patient-Rated Wrist/Hand Evaluation (PRWHE) for patients with osteoarthritis at the base of thumb
Source: BMC Med Res Methodol. 2022 Apr 29;22:127. doi: 10.1186/s12874-022-01600-1 (PMC9052459; doi:10.1186/s12874-022-01600-1)
Supplement: Supplementary file 1 — Additional file 1: Table S1. Correlations between post scores and GRC. Table S2. Correlations between GRC and the change of the target instrument. Table S3. Correlations between GRC and target instrument baseline scores. [file 12874_2022_1600_MOESM1_ESM.pdf]

Supplementary appendices:

Table S1 Correlations between post scores and GRC

Time points	Pain intensity (NRS)	PRWHE pain	PRWHE function	PRWHE total
6 weeks	0.20(-0.07 to 0.44)	0.32*(0.05 to 0.54)	0.26(-0.02 to 0.55)	0.31*(0.03 to 0.55)
3 months	0.64**(0.44 to 0.80)	0.51**(0.28 to 0.71)	0.37**(0.07 to 0.64)	0.45**(0.20 to 0.70)
6 months	0.54**(0.30 to 0.72)	0.54**(0.28 to 0.72)	0.55**(0.32 to 0.73)	0.54**(0.31 to 0.73)
9 months	0.46**(0.20 to 0.68)	0.48**(0.15 to 0.69)	0.52**(0.18 to 0.73)	0.52**(0.19 to 0.73)
12 months	0.51**(0.28 to 0.68)	0.39**(0.04 to 0.62)	0.39**(0.19 to 0.59)	0.45**(0.25 to 0.62)
All time points	0.72**(0.64 to 0.78)	0.73**(0.67 to 0.79)	0.69**(0.61 to 0.75)	0.72**(0.66 to 0.78)

Values are Spearman's rho with 95 % CI. * p < 0.05. ** p < 0.01.

Table S2 Correlations between GRC and the change of the target instrument

Time points	Pain intensity (NRS)	PRWHE pain	PRWHE function	PRWHE total
6 weeks	-0.16(-0.42 to 0.13)	-0.11(-0.41 to 0.21)	-0.030(-0.33 to 0.29)	-0.14(-0.43 to 0.19)
3 months	-0.62**(-0.80 to -0.40)	-0.64**(-0.81 to -0.42)	-0.61**(-0.78 to -0.39)	-0.72**(-0.86 to -0.52
6 months	-0.49**(-0.68 to -0.25)	-0.63**(-0.77 to -0.41)	-0.63**(-0.78 to -0.44)	-0.66**(-0.79 to -0.48
9 months	-0.27(-0.51 to -0.02)	-0.35*(-0.59 to -0.07)	-0.49**(-0.66 to -0.25)	-0.47**(-0.65 to -0.23
12 months	-0.24(-0.50 to 0.10)	-0.22(-0.47 to 0.12)	-0.26(-0.47 to -0.03)	-0.26(-0.46 to -0.02)
All time points	-0.55**(-0.64 to -0.45)	-0.71**(-0.77 to -0.64)	-0.70**(-0.76 to -0.63)	-0.74**(-0.80 to -0.68

Values are Spearman's rho with 95 % CI. * p < 0.05. ** p < 0.01.

Table S3 Correlations between GRC and target instrument baseline scores

Time points	Pain intensity (NRS)	PRWHE pain	PRWHE function	PRWHE total
GRC at	Baseline			
6 weeks	0.11(-0.17 to 0.41)	0.21(-0.06 to 0.48)	0.18(-0.12 to 0.45)	0.21(-0.08 to 0.5)
3 months	-0.080(-0.38 to 0.21)	-0.21(-0.47 to 0.03)	-0.26(-0.56 to 0.02)	-0.30*(-0.57 to -0.03)
6 months	-0.14(-0.41 to 0.11)	-0.19(-0.46 to 0.04)	-0.14(-0.44 to 0.13)	-0.23(-0.52 to 0.02)
9 months	0.22(0.02 to 0.47)	0.18(-0.03 to 0.42)	-0.030(-0.34 to 0.28)	0.040(-0.26 to 0.32)
12 months	0.19(-0.01 to 0.42)	0.23(0.01 to 0.47)	0.040(-0.23 to 0.38)	0.11(-0.15 to 0.43)

Values are Spearman's rho with 95 % CI. * p < 0.05. ** p < 0.01.
